# Author Correction: Downstream high-speed plasma jet generation as a direct consequence of shock reformation

**DOI:** 10.1038/s41467-022-28664-3

**Published:** 2022-02-16

**Authors:** Savvas Raptis, Tomas Karlsson, Andris Vaivads, Craig Pollock, Ferdinand Plaschke, Andreas Johlander, Henriette Trollvik, Per-Arne Lindqvist

**Affiliations:** 1 Division of Space and Plasma Physics - KTH Royal Institute of Technology, Stockholm, Sweden; 2 Denali Scientific, Fairbanks, AK 99709 USA; 3 Institute of Geophysics and Extraterrestrial Physics, Technische Universität Braunschweig, Brunswick, Germany; 4 Space Research Institute, Austrian Academy of Sciences, Graz, Austria; 5 Department of Physics, University of Helsinki, Helsinki, Finland; 6 Swedish Institute of Space Physics, Uppsala, Sweden

**Keywords:** Astrophysical plasmas, Magnetospheric physics, Space physics

Correction to: *Nature Communications* 10.1038/s41467-022-28110-4, published online 1 February 2022.

In this article the affiliation Space Research Institute, Austrian Academy of Sciences, Graz, Austria for Author Ferdinand Plaschke was missing. Author name Savvas Raptis was incorrectly written as Savvas Rapitis. The original article has been corrected.

